# A Prospective Study of Fruit Juice Consumption and the Risk of Overall and Cardiovascular Disease Mortality

**DOI:** 10.3390/nu14102127

**Published:** 2022-05-19

**Authors:** Zhuang Zhang, Xueke Zeng, Meiling Li, Tengfei Zhang, Haowei Li, Hu Yang, Yong Huang, Yu Zhu, Xiude Li, Wanshui Yang

**Affiliations:** 1 Department of Nutrition, School of Public Health, Anhui Medical University, Hefei 230032, China; 2020500011@ahmu.edu.cn (Z.Z.); 2045010290@stu.ahmu.edu.cn (X.Z.); 2045010416@stu.ahmu.edu.cn (M.L.); 2045010404@stu.ahmu.edu.cn (T.Z.); 2013260044@stu.ahmu.edu.cn (H.L.); 2045010405@stu.ahmu.edu.cn (H.Y.); 2146010036@stu.ahmu.edu.cn (Y.H.); 2046010043@stu.ahmu.edu.cn (Y.Z.); 2046010029@stu.ahmu.edu.cn (X.L.); 2 Key Laboratory of Population Health Across Life Cycle, Anhui Medical University, Ministry of Education of the People’s Republic of China, Hefei 230032, China; 3 NHC Key Laboratory of Study on Abnormal Gametes and Reproductive Tract, Hefei 230032, China; 4 Anhui Provincial Key Laboratory of Population Health and Aristogenics/Key Laboratory of Environmental Toxicology of Anhui Higher Education Institutes, Anhui Medical University, Hefei 230032, China; 5 Anhui Provincial Institute of Translational Medicine, Hefei 230032, China

**Keywords:** fruit juice, fruit, mortality, cardiovascular disease, substitution analysis

## Abstract

There is little evidence for the association between fruit juice, especially 100% fruit juice, and mortality risk. In addition, whether 100% fruit juice can be a healthy alternative to whole fruit remains uncertain. This prospective study utilized the data from the US National Health and Nutrition Examination Survey (NHANES) from 1999 to 2014. After a median follow-up of 7.8 years, 4904 deaths among 40,074 participants aged 18 years or older were documented. Compared to non-consumption, daily consumption of 250 g or more of 100% fruit juice was associated with higher overall mortality (hazard ratio (HR) = 1.30, 95% confidence interval (CI): 1.11–1.52) and mortality from heart disease (HR = 1.49, 95 CI: 1.01–2.21). A similar pattern was observed for total fruit juice, with HRs of 1.28 (95% CI: 1.09–1.49) for overall mortality and 1.48 (95% CI: 1.01–2.17) for heart disease mortality. Replacing 5% of energy from whole fruit with 100% or total fruit juice was associated with a 9% (95% CI: 2–16%) and 8% (95% CI: 1–15%) increased mortality risk, respectively. Our findings suggest that both total and 100% fruit juice could be associated with high mortality risk, and need to be validated in well-designed studies given the potential misclassification of diet and death reasons.

## 1. Introduction

It is well acknowledged that the consumption of whole fruit benefits health [1,2], but the evidence pertaining to the effects of fruit juice, especially 100% fruit juice, is limited and debatable [3,4,5]. Current dietary recommendations for 100% fruit juice consumption differ across countries. The dietary guidelines from the United States, United Kingdom, and France all state that the recommended daily fruit intake can be partly replaced by 100% fruit juice [6,7,8]. In contrast, Dutch and Italian guidelines keep a more prudent attitude toward 100% fruit juice due to its high content of natural sugars being similar to the sugar content of sugar-sweetened beverages (SSBs), and thus suggest minimizing consumption [9,10]. Therefore, more evidence for the health effects of fruit juice is needed. In addition, whether 100% fruit juice can be a healthy alternative to whole fruit remains to be further investigated.

However, few epidemiological studies have examined the associations between fruit juice and mortality risk, yielding an inverse [11], null [12,13], and a positive association [14]. In addition, the above-mentioned studies did not consider the non-linear association, which cannot be ruled out in the actual relation of fruit juice intake and death risk [15]. Moreover, no study, to our knowledge, has assessed the associations between replacing whole fruit with 100% or total fruit juice and mortality risk.

To fill in the knowledge gap, we aimed to prospectively investigate the associations between fruit juice consumption and the risk of overall and CVD mortality among US adults, allowing for the potential linear and non-linear relationships. Additionally, we performed a substitution analysis to evaluate the associations between isocalorically replacing whole fruit consumption with 100% or total fruit juice and death risk.

## 2. Materials and Methods

### 2.1. Study Population

The National Health and Nutrition Examination Survey (NHANES) consists of a series of continuous cross-sectional surveys of the civilian, noninstitutionalized US population since 1999, with a complex, stratified, multistage probability sampling design. It combines personal interviews with standardized physical examinations and laboratory tests administered by a specially trained staff that travels to selected survey sites to collect data on a nationally representative sample of the US population. Details of the NHANES study protocol and data collection methods have been reported elsewhere [16].

For the present study, participants of the NHANES survey cycles from 1999–2000 to 2013–2014 were included, since the mortality data of the participants were renewed until 2015. We excluded the participants who were younger than 18 years old (*n* = 34,735), had anomalous energy intake (<600 or >3500 kcal/day for women and <800 or >4200 kcal/day for men, *n* = 2100), had missing dietary data (*n* = 5132), or did not have linked mortality data (*n* = 50). Therefore, a total of 40,074 participants were included in the final analysis (Figure S1).

### 2.2. Dietary Assessment

After obtaining informed consent, participants were scheduled for a mobile examination center (MEC) visit. Dietary data were collected using a 24-h dietary recall administered by trained interviewers in the MEC using the Automated Multiple-Pass Method (AMPM). The AMPM is a computer-assisted multiple-pass format interview system with standardized probes, developed by the USDA to estimate current dietary intake and to minimize misreporting [17]. From 1999 to 2002, only a single 24-h dietary recall was performed. Since 2003, an additional 24-h dietary recall was conducted 3–10 days after the first recall to account for day-to-day variation.

The definition of 100% fruit juice is described in Table S1. Total fruit juice intake was defined as the sum of all kinds of fruit juice, including fruit juice added with free sugars or sweeteners (Table S2). For the participants with two 24-h dietary recalls (*n* = 27,580, 68.8%), the averages of the intake amounts were used, otherwise the intake amounts from the single 24-h dietary recall (*n* = 12,494, 31.2%) were used.

### 2.3. Assessment of Covariates

Information on covariates was collected through questionnaires, administrated during the household interview, that included demographic and lifestyle factors (i.e., age, sex, race/ethnicity, educational level, family income, physical activity, and smoking status). Information on body weight, height, and alcohol drinking status was obtained during the MEC visit. The body mass index (BMI) was calculated as weight (kg) divided by the square of height (m^2^). The income-to-poverty ratio (IPR) was used as the measure of economic status, calculated by dividing the total family income by the poverty threshold. Histories of diabetes, hypertension, other CVDs, and cancer were defined according to self-reported medical diagnoses of these diseases and use of prescribed medications due to these diseases. The participants with a fasting glucose of 126 mg/dL or greater were also defined as diabetic patients. Hypertension (a systolic blood pressure ≥140 mmHg or a diastolic blood pressure ≥90 mmHg) was also identified through physical examination in the MEC. Dyslipidemia was defined according to the methods by Huang et al. [18].

### 2.4. Ascertainment of Deaths

We ascertained mortality status via record linkage to the National Death Index (NDI) through 31 December 2015. In our analysis, CVD mortality was defined using the 10th revision of the International Classification of Diseases (ICD-10), including deaths from diseases of the heart (ICD-10 codes I00-I09, I11, I13, I20-I51) and cerebrovascular diseases (I60–169). The NDI has been proven to be a reliable and efficient utility for ascertainment of deaths in large epidemiological studies, and over 98% of deaths can be identified using this approach [19,20].

### 2.5. Statistical Analysis

Person-years were calculated from the baseline to the date of death, loss to follow-up, or 31 December 2015, whichever came first. After rounding, both 100% fruit juice and total fruit juice consumption were categorized as 0, 1–124, 125–249, and 250 or more (g/day). These cut-off values provide relatively equal sample sizes for each exposure group among fruit juice consumers in the present analysis. We used Cox proportional hazards regression models to calculate hazard ratios (HRs) and 95% confidence intervals (CIs) of death according to fruit juice consumption categories. HRs of death risk for each 100 g/day increase in fruit juice intake was also calculated. Sampling weights were used to enable the study population to better represent the entire national population. Model 1 was adjusted for sex, age, and total energy intake. Model 2 was further adjusted for race/ethnicity, education, marital status, ratio of family income to poverty, physical activity, smoking status, drinking status, body mass index, diabetes, cancer, dyslipidemia, hypertension, other CVDs (without hypertension), and the Healthy Eating Index-2015 (HEI-2015). To avoid over-adjustment, the intake component of 100% fruit juice was removed from the HEI-2015 score. Restricted cubic splines with 3 knots were used to test the potential non-linear relationship between fruit juice intake and death risk. We also investigated the association between two specific 100% fruit juices (i.e., orange juice and citrus juice) and mortality risk. Due to relatively low intake levels, we only reported HRs and 95% CIs for each 100 g/day increase in the individual 100% fruit juice.

The substitution analysis was performed to evaluate whether replacement of whole fruit with fruit juice is beneficial or deleterious for health. We used the leave-one-out model [21] to investigate the associations between the isocaloric replacement of 5% of energy from whole fruit with the equivalent energy from total or 100% fruit juice and mortality risk.

A subgroup analysis was conducted by age, sex, race/ethnicity, education level, IPR, marital status, smoking status, alcohol drinking status, physical activity level, BMI, and history of diabetes. We used the Wald test to examine whether the interaction (i.e., cross-product terms) between these variables and exposures were statistically significant. In a sensitivity analysis, we repeated the analysis by excluding individuals with only a single 24-h dietary recall interview. To reduce the reverse causation, we also repeated the analysis by excluding deaths that occurred within 3 years after the first dietary recall. Considering that participants may change their dietary habits due to their health conditions, we performed an analysis on participants who did not have a history of diabetes, cancer, or major CVDs (i.e., congestive heart failure, angina pectoris, coronary heart disease, heart attack, and stroke) at the baseline. All *p*-values are 2-sided at a type I error rate of 0.05. All statistical tests were performed using SAS version 9.4 (SAS Institute Inc, Cary, NC, USA).

## 3. Results

### 3.1. Baseline Characteristics

After following 40,074 participants (mean age, 47.3 years; SD, 19.4 years) for 325,905 person-years (median follow-up time, 7.8 years), 4904 deaths, including 1029 CVD-specific deaths, were documented. Among participants who reported consuming 100% or total fruit juice, their median intake levels of 100% or total fruit juice were 182 (interquartile range (IQR): 101–299) g/day and 186 (IQR: 108–311) g/day, respectively. Participants consuming more 100% fruit juice were less likely to be non-Hispanic white, had a higher energy intake, adhered to the HEI-2015 (without the intake component of fruit juice), and were more physically active (Table 1).

### 3.2. Association between Fruit Juice Consumption and Mortality

In general, higher consumption of 100% and total fruit juice was associated with increased risk of all-cause mortality and heart disease-specific mortality, but not with cerebrovascular disease-specific mortality (Table 2). In the fully adjusted model, the HR of overall mortality was 1.30 (95% CI: 1.11–1.52, *p*_trend_ < 0.001) for those who consumed 250 g/day or more of 100% fruit juice, compared with non-consumers. Each 100 g/day increase in 100% fruit juice consumption was associated with a 6% (95% CI: 3–9%) increased risk of all-cause mortality. We found a significant association between 100% fruit juice and heart disease-specific mortality (HR = 1.49, 95% CI: 1.01–2.21, *p*_trend_ = 0.026), but not with cerebrovascular disease-specific mortality (HR = 0.89, 95% CI: 0.30–2.62, *p*_trend_ = 0.518).

When assessing the specific 100% fruit juice, each 100 g/day increase in orange juice and citrus juice generally showed a positive association with mortality risk, with HRs of 1.07 (95% CI: 1.02–1.11) and 1.07 (95% CI: 1.02–1.11) for total mortality (Table S3).

When assessing total fruit juice, the HRs (comparing daily consumption of 250 g or more of total fruit juice with no consumption) were 1.28 (95% CI: 1.09–1.49, *p*_trend_ < 0.001) for overall mortality, 1.48 (95% CI: 1.01–2.17, *p*_trend_ = 0.023) for heart disease-specific mortality, and 0.83 (95% CI: 0.28–2.42, *p*_trend_ = 0.399) for cerebrovascular disease-specific mortality.

**Figure 1:**
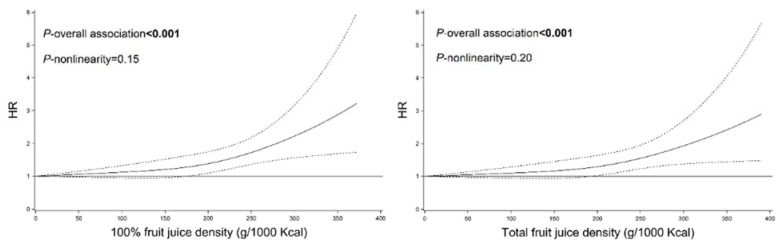
Associations between fruit juice and overall mortality in NHANES (1999–2014). HRs—hazard ratios; NHANES—National Health and Nutrition Examination Survey. Covariates adjusted in the models were the same as those in Model 2 in Table 2 (see Table 2 footnote).

A restricted multivariable cubic spline analysis did not support a non-linear association between 100% or total fruit juice intake and mortality risk (Figure 1), as all *p*-values for non-linearity were greater than 0.05.

### 3.3. Substitution Analysis

In isocalorical models (Table 3), the replacement of 5% of energy intake from whole fruit with an equivalent energy intake from 100% fruit juice was associated with an increased risk of overall mortality (HR = 1.09, 95% CI:1.02–1.16), but not with CVD mortality (HR = 1.00, 95% CI:0.88–1.15). Similarly, the substitution of total fruit juice for whole fruit was associated with an 8% (95% CI:1–15%) elevated risk of all-cause mortality, but not with CVD mortality risk.

### 3.4. Secondary Analysis

**Figure 2:**
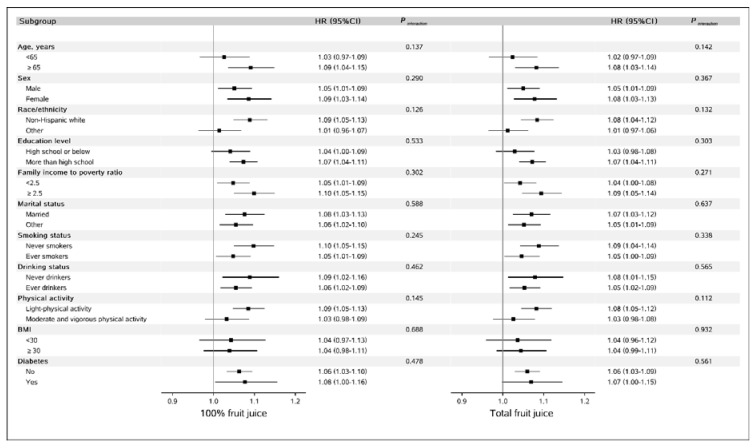
Subgroup analysis on the association between fruit juice (per 100 g/day increase) and all-cause mortality in NHANES (1999–2014) BMI—body mass index; CIs—confidence intervals; HRs—hazard ratios; METS—metabolic equivalent tasks; NHANES—National Health and Nutrition Examination Survey. Covariates adjusted in the models were the same as those in Model 2 in Table 2 (see Table 2 footnote). Of note, variables examined in this figure were not adjusted. Light physical activity was defined as participants with physical activity less than 8.3 METS-h per week, and moderate and vigorous activity was defined as participants who had physical activity of 8.3 METS-h per week or more.

In the subgroup analysis, we did not find any significant differential association between fruit juice intake and risk of overall mortality (Figure 2) according to age, sex, race/ethnicity, education level, IPR, marital status, smoking, alcohol drinking, physical activity, BMI, or diabetes (all *p*-values for interactions were greater than 0.05).

In the sensitivity analysis, after excluding individuals who received only one dietary interview (*n* = 12,494, 31.2%), individuals who passed away within 3 years after the first dietary recall (*n* = 1308, 3.3%), or individuals who had a history of diabetes, cancer, or CVD at the baseline (*n* = 6716, 16.8%), the results were not essentially changed (Table S4).

## 4. Discussion

In this prospective study of 40,074 nationally representative US adults, both 100% and total fruit juice consumption could be associated with a higher risk of overall mortality and mortality from heart diseases. In addition, substituting 100% or total fruit juice for whole fruit might be associated with increased mortality risk. These findings add novel evidence to the ongoing debate on the potential long-term health effects of 100% fruit juice, suggesting more caution when considering 100% fruit juice as the alternative to whole fruit in daily food selection.

Previous studies of fruit juice and all-cause mortality are limited and have yielded inconsistent results [11,12,13,14]. A prospective cohort study utilizing the data of 198,285 UK Biobank participants (aged 40–69 years) demonstrated an apparent inverse dose–response relationship of fruit or vegetable juice with all-cause mortality [11], whereas the association did not persist after additional adjustment for a diet quality score. However, in the present study, we did not observe such inverse associations in the model without adjustment for HEI-2015. The discrepancies could be possibly due to the inclusion of vegetable juice in the UK Biobank study. In another prospective study of 52,584 Chinese participants (aged 45–74 years), a null association was found between the intake frequency of fruit or vegetable juice and all-cause or specific-cause mortality [13]. A different quantitation method of juice consumption could partly explain the discrepancies. In accordance with our results, a US cohort study of 13,440 participants aged 45 years or older (mean age: 63.6 years), the Reasons for Geographic and Racial Differences in Stroke (REGARDS) Study, reported a 24% (9–42%) increased risk of total mortality for each additional 12 oz of 100% fruit juice consumption [14]. Our findings extend the associations between 100% fruit juice consumption and enhanced all-cause and heart disease-specific mortality risk in a nationally representative sample of US adults aged 18 years and older.

Similarly, epidemiological evidence regarding the associations of 100% fruit juice consumption and CVD mortality or incidence remains controversial [12,14,22,23,24,25]. Consistent with our results, most studies showed a non-significant association between fruit juice and CVD mortality, including results of the REGARDS cohort [14], the Nurses’ Health Study and the Health Professionals Follow-up Study [22], and the Singapore Chinese Health Study [13], though in the Singapore Chinese Health Study 100% fruit juice was not distinguished from total fruit juice. In a recent meta-analysis of 21 prospective studies and 35 randomized controlled trials, higher consumption of 100% fruit juice is not associated with cardiovascular risk (e.g., blood pressure, lipid profile, glucose homeostasis), whereas low to moderate intake of 100% fruit juice was inversely associated with the risk of incident stroke and total CVDs [24]. In addition, studies have shown a non-linear relationship between 100% fruit juice and CVD incidence, revealing a protective effect at moderate doses (~80 mL/day [24] or less than 150 mL/day [15]) but indicating harm at higher doses [15]. However, in the present study, our results did not support a non-linear association between 100% fruit juice intake and all-cause or CVD-specific mortality. The conflicting results can be partly explained by different dietary assessment methods, different confounders adjusted for in these studies, and different dietary habits among diverse populations. More prospective studies with larger sample sizes are still needed to explore the dose–response relationship between 100% fruit juice consumption and mortality risk.

The conflicting results are reflected in the inconsistencies among different dietary guidelines about the recommendations for the proper status of 100% fruit juice. The major contradiction lies on whether 100% fruit juice can be considered as an acceptable alternative to whole fruit. All international guidelines recommend consuming enough fruit [26,27], but only a small minority of the population consumes the recommended amount of fruit and vegetables due to factors related to practicalities, convenience, and the effort required [28,29]. Fruit juice is convenient, easily transportable, and requires no preparation, offering a solution to many of these problems. Hence, 100% fruit juice counts as a part of the recommended fruit per day in some countries [7,8,29]. The dietary guidelines from the UK state that 100% fruit juice can contribute one portion to the daily fruit portion size of 150 mL [7]. The French guidelines also suggest that fruit juice can count as one portion of fruit and vegetables per day, but the consumption should be limited to less than one glass/day [8]. The Dietary Guidelines for Americans (GDA) 2020–2025 states that less than half of the recommended daily fruit intake can be replaced by 100% fruit juice, but daily intake of 100% fruit juice should be restricted due to the lower dietary fiber content when compared to whole fruit [6]. Other dietary guidelines have claimed that fruit juice is little more than a source of sugar and have proposed that fruit juice should not be included in daily fruit consumption [30,31]. The Dutch guidelines classify fruit juice as SSBs; thus, they suggest minimizing the consumption [9]. The Italian guidelines also consider fruit juice as unnecessary consumption [10]. The World Health Organization (WHO) recommends reducing the intake of free sugars, including both added sugars and sugars naturally present in fruit juice, to less than 10% (and, ideally, less than 5%) of the total daily energy intake [32]. The method of substitution analysis enables us to address such contradictions. According to our results from the substitution analysis, replacing a part of daily whole fruit consumption with 100% fruit juice is possibly not a healthy choice. Our findings support the recommendations to limit the intake of fruit juice, including 100% fruit juice. Still, more further studies are needed to confirm our findings.

There are several plausible biological mechanisms to explain the elevated mortality risk that comes with higher 100% fruit juice consumption. Firstly, 100% fruit juice usually contains a similar amount of sugar as SSBs, and end up with similar energy densities [15]. Compared with whole fruit, fruit juice provides much less satiety, leading to passive energy hyperconsumption and weight gain [33]. Therefore, the effect of overweight and obesity onset could partly explain these associations. However, in this study, BMI and energy intake were adjusted for our full model, and stratification by baseline BMI status presented similar results. Moreover, 100% fruit juice presented a similar pattern to that of total fruit juice in the associations with death risk. It seems that it is the sugar itself in the fruit juice, which is mostly fructose, rather than excess energy intake that possibly cause the adverse health effect. It has been suggested that fructose might enhance lipogenesis, worsen blood lipids, and promote visceral adiposity independently of body weight [34,35,36,37], causing a long-term all-cause death risk. In addition, the high glycemic index or glycemic load of fruit juice consumption is reported to be related to a higher systemic inflammation, which is a risk factor for many chronic diseases [38]. Apart from the rich liquid fructose, fruit juice contains much less fiber than whole fruit; this can also be related to the hazardous effect of replacing whole fruit with 100% fruit juice. A dietary crossover study demonstrated that intake of both whole apples and cloudy apple juice can reduce serum LDL concentration, but clear apple juice increased serum LDL concentration, revealing the necessary role of the fiber component in the cholesterol-lowering effect of fruit [39]. These findings also give a hint that distinguishing cloudy juice from clear juice in further studies will help to better access the intrinsic effects of different types of juice. Although the nutrients appear as the main driver of the association, other chemical compounds, such as pesticides or additives in the bottled fruit juice products, might also be causal factors [40,41].

This study has several strengths, including the use of a nationally representative sample of US adults, large sample size, and a prospective cohort design. There are also several limitations. First, the dietary information was collected by the 24-h dietary recall, which can cause misclassification of sugar-sweetened juice as 100% fruit juice, although such misclassification is also inevitable in other dietary assessment methods such as the food frequency questionnaire and dietary record [42]. Second, dietary information was collected based on the baseline measurement, but the participants may change their dietary habits during the follow-up. Third, although we have adjusted for a wide range of risk factors, the possibility of residual confounding variables, such as individuals’ gene variants and metabolic status, cannot be totally ruled out. Fourth, despite a nationally representative sample in the study, our findings might not be generalizable to other populations (e.g., Asian populations). Therefore, studies in diverse populations are warranted to refute or replicate our results.

## 5. Conclusions

Our study found that consumption of 100% or total fruit juice could be associated with a higher risk of mortality, and substituting 100% or total fruit juice for whole fruit might be associated with an increased death risk. However, these results should be interpreted with caution, given the single measurement of diet using 24-h recalls and the death ascertainment using the NDI, which may lead to misclassifications of fruit juices and death reasons. Further studies with a larger sample size, careful consideration of fruit juice type (clear or cloudy), and other possible confounders are warranted to confirm our findings and to shape evidence-based dietary guidelines.

## Figures and Tables

**Table 1:** Age-adjusted characteristics of participants according to 100% fruit juice consumption in NHANES (1999–2014) ^a^.

Characteristic	100% Fruit Juice (g/day)			
	0	1 to 124	125 to 249	≥250
No. of participants	27,032	4782	4156	4104
Age, years	46.7 (18.9)	51.5 (19.4)	50.7 (20.9)	43.4 (19.7)
Female, %	51.5	61.0	54.3	46.1
BMI, kg/m^2^	28.7 (6.8)	28.4 (6.5)	28.3 (6.5)	28.3 (6.5)
Race/ethnicity, %				
Non-Hispanic white	48.1	41.9	44.1	38.3
Non-Hispanic black	19.9	19.8	23.5	26.1
Hispanic	6.7	8.8	8.5	8.8
Other	25.4	29.5	23.9	26.8
Education, %				
≤12th grade	30.1	26.0	25.1	29.7
High school graduate/GED or equivalent	24.7	21.9	22.9	21.6
More than high school	45.1	52.0	52.0	48.6
Ratio of family income to poverty				
<1.3	29.8	27.2	27.4	30.0
1.3 to 3.5	34.6	34.6	33.6	33.4
≥3.5	27.8	30.1	30.5	28.5
Marital status, %				
Married	26.3	23.3	26.1	28.2
Widowed/divorced/separated	52.3	55.6	53.1	50.3
Never married	17.6	17.6	16.9	17.4
Smoking, %				
Never smoking	47.7	58.1	55.4	53.8
Former smoking	24.1	22.1	24.1	21.6
Current smoking	21.6	14.3	14.0	15.7
Drinking, %				
Never drinking	26.0	28.7	28.0	27.4
Low to moderate drinking	26.3	27.7	27.4	25.3
Heavy drinking	36.4	33.1	33.7	33.2
Physical activity, METS-h/week				
<8.3	41.2	40.7	39.4	38.3
8.3–16.7	12.3	12.6	12.1	11.7
>16.7	46.2	46.4	48.3	49.6
Total energy, kcal/d	1975 (734)	1955 (695)	2049 (712)	2237 (737)
History of diseases, %				
Diabetes	13.2	12.0	10.0	10.1
Other CVDs (without hypertension)	10.4	9.2	10.6	10.0
Cancer	8.4	9.1	9.4	7.6
Hypertension	35.7	35.2	35.8	33.3
Dyslipidemia	56.5	54.0	54.1	57.1
HEI-2015 (without fruit juice component)	48.7 (11.9)	52.2 (11.9)	52.6 (11.5)	53.6 (11.1)

BMI—body mass index; CIs—confidence intervals; CVD—cardiovascular diseases; GED—general educational development; HEI-2015—Healthy Eating Index-2015; METS—metabolic equivalent tasks; NHANES—National Health and Nutrition Examination Survey; SD—standard deviation. Of note, the intake component of fruit juice was removed from the HEI-2015; ^a^ variables were adjusted for age. Continuous variables were expressed as mean (SD) if normally distributed. Categorical variables were expressed as proportion (%). Values of polytomous variables may not sum to 100% due to missing values or rounding.

**Table 2:** HRs (95% CIs) for mortality risk according to fruit juice consumption (g/day) in NHANES (1999–2014).

Fruit Juice Consumption	HR (95% CI)					*P*_trend_^c^
(g/day)	0	1 to 124	125 to 249	≥250	Per 100 g/day Increase	
100% fruit juice						
All-cause mortality						
No. of deaths/person-years	3180/222,833	630/34,656	649/32,387	445/36,029		
Model 1 ^a^	1 (Reference)	1.01 (0.89–1.16)	1.06 (0.92–1.22)	1.13 (0.94–1.35)	1.04 (0.99–1.08)	0.109
Model 2 ^b^	1 (Reference)	1.15 (1.00–1.31)	1.17 (0.99–1.38)	1.30 (1.11–1.52)	1.07 (1.04–1.10)	<0.001
CVD mortality						
No. of deaths/person-years	677/222,833	117/34,656	147/32,387	88/36,029		
Model 1 ^a^	1 (Reference)	0.74 (0.55–0.99)	0.91 (0.69–1.21)	1.13 (0.73–1.74)	1.03 (0.92–1.15)	0.633
Model 2 ^b^	1 (Reference)	0.87 (0.65–1.15)	1.04 (0.74–1.45)	1.38 (0.93–2.06)	1.07 (0.99–1.16)	0.093
Heart disease mortality						
No. of deaths/person-years	557/222,833	94/34,656	124/32,387	71/36,029		
Model 1 ^a^	1 (Reference)	0.75 (0.55–1.02)	0.94 (0.70–1.26)	1.20 (0.77–1.88)	1.05 (0.93–1.18)	0.471
Model 2 ^b^	1 (Reference)	0.88 (0.65–1.20)	1.07 (0.76–1.51)	1.49 (1.01–2.21)	1.09 (1.01–1.18)	0.026
Cerebrovascular disease mortality						
No. of deaths/person-years	120/222,833	23/34,656	23/32,387	17/36,029		
Model 1 ^a^	1 (Reference)	0.71 (0.29–1.77)	0.80 (0.34–1.85)	0.76 (0.26–2.22)	0.88 (0.67–1.16)	0.371
Model 2 ^b^	1 (Reference)	0.79 (0.30–2.05)	0.89 (0.40–1.96)	0.89 (0.30–2.62)	0.92 (0.71–1.19)	0.518
Total fruit juice						
All-cause mortality						
No. of deaths/person-years	3117/217,026	631/34,829	670/34,056	486/39,995		
Model 1 ^a^	1 (Reference)	1.01 (0.89–1.15)	1.01 (0.89–1.16)	1.09 (0.91–1.31)	1.03 (0.99–1.07)	0.193
Model 2 ^b^	1 (Reference)	1.14 (1.00–1.30)	1.15 (0.98–1.34)	1.28 (1.09–1.49)	1.06 (1.03–1.09)	<0.001
CVD mortality						
No. of deaths/person-years	658/217,026	122/34,829	153/34,056	96/39,995		
Model 1 ^a^	1 (Reference)	0.84 (0.64–1.11)	0.92 (0.71–1.21)	1.09 (0.71–1.68)	1.02 (0.92–1.14)	0.709
Model 2 ^b^	1 (Reference)	0.98 (0.75–1.28)	1.07 (0.78–1.47)	1.36 (0.92–2.01)	1.07 (0.99–1.16)	0.101
Heart disease mortality						
No. of deaths/person-years	542/217,026	98/34,829	129/34,056	77/39,995		
Model 1 ^a^	1 (Reference)	0.87 (0.63–1.19)	0.97 (0.73–1.28)	1.16 (0.75–1.81)	1.04 (0.93–1.17)	0.518
Model 2 ^b^	1 (Reference)	1.01 (0.73–1.40)	1.12 (0.81–1.56)	1.48 (1.01–2.17)	1.09 (1.01–1.17)	0.023
Cerebrovascular disease mortality						
No. of deaths/person-years	116/217,026	24/34,829	24/34,056	19/39,995		
Model 1 ^a^	1 (Reference)	0.76 (0.32–1.81)	0.75 (0.32–1.73)	0.71 (0.24–2.06)	0.86 (0.66–1.14)	0.292
Model 2 ^b^	1 (Reference)	0.84 (0.34–2.07)	0.81 (0.38–1.73)	0.83 (0.28–2.42)	0.90 (0.69–1.16)	0.399

BMI—body mass index; CIs—confidence intervals; CVD—cardiovascular diseases; GED—general educational development; HEI-2015—Healthy Eating Index-2015; HRs—hazard ratios; METS—metabolic equivalent tasks; NHANES—National Health and Nutrition Examination Survey. ^a^ Model 1 was adjusted for sex (male, female), age (18–45, 46–65, and ≥66 years), and total energy intake (kcal/day, tertile); ^b^ Model 2 was further adjusted for race/ethnicity (non-Hispanic white, non-Hispanic black, Hispanic, and other), education (≤12th grade, high school graduate/GED or equivalent, and more than high school), marital status (married, widowed/divorced/separated, and never married), ratio of family income to poverty (<1.30, 1.30–3.49, and ≥3.50), physical activity (<8.3, 8.3–16.7, and >16.7 METS h/week), smoking (never smoking, former smoking, and current smoking), drinking (never drinking, low to moderate drinking, and heavy drinking), BMI (<18.5, 18.5–24.9, 25.0–29.9, and 30.0 kg/m^2^), diabetes (no, yes), baseline of cancer (no, yes), dyslipidemia (no, yes), hypertension (no, yes), other CVDs (no, yes), and HEI-2015 (score, tertile). Of note, the intake component of fruit juice was removed from HEI-2015 to avoid over-adjustment in the multivariable-adjusted models. ^c^ Linear trend test was conducted by treating 100% or total fruit juice as continuous variable in the model.

**Table 3:** HRs (95% CIs) for isocalorical replacement of whole fruit with fruit juice in NHANES (1999–2014) ^a^.

	HR (95% CI)	
Cause of Death	100% Fruit Juice	Total Fruit Juice
All-cause mortality		
Model 1 ^b^	1.18 (1.10–1.27)	1.16 (1.08–1.25)
Model 2 ^c^	1.09 (1.02–1.16)	1.08 (1.01–1.15)
CVD mortality		
Model 1 ^b^	1.03 (0.91–1.17)	1.01 (0.89–1.15)
Model 2 ^c^	1.00 (0.88–1.15)	0.99 (0.87–1.13)

CIs—confidence intervals; CVD—cardiovascular diseases; HRs—hazard ratios; NHANES—National Health and Nutrition Examination Survey. ^a^ HRs were calculated as the mortality risk for isocaloric replacement of 5% of energy from whole fruit with equivalent energy from total or 100% fruit juice; ^b^ covariates adjusted in Model 1 were the same as those in Model 1 in Table 2 (see Table 2 footnote); ^c^ covariates adjusted in Model 2 were the same as those in Model 2 in Table 2 (see Table 2 footnote).

## Data Availability

Data described in the manuscript, code book, and analytic code will be made publicly and freely available without restriction (Available online: https://www.cdc.gov/nchs/nhanes/index.htm, accessed on 5 September 2021).

## Supplementary Materials

The following are available online at https://www.mdpi.com/article/10.3390/nu14102127/s1, Figure S1: Flow chart of selection of participants in this analysis; Table S1: Food codes of 100% fruit juice in NHANES (1999–2014); Table S2: Food codes of total fruit juice in NHANES (1999–2014); Table S3: HRs (95% CIs) for mortality risk according to each 100 g/day increase in intake of 100% orange juice and 100% citrus juice in NHANES (1999–2014); Table S4: Sensitivity analyses on association between consumption of fruit juice with the risk of all-cause mortality in NHANES (1999–2014).
