# Supplementary material for: A Prospective Study of Fruit Juice Consumption and the Risk of Overall and Cardiovascular Disease Mortality
Source: Nutrients. 2022 May 19;14(10):2127. doi: 10.3390/nu14102127 (PMC9144949; doi:10.3390/nu14102127)
Supplement: Supplementary file 1 [file nutrients-14-02127-s001.zip › nutrients-1704803-supplementary.pdf]

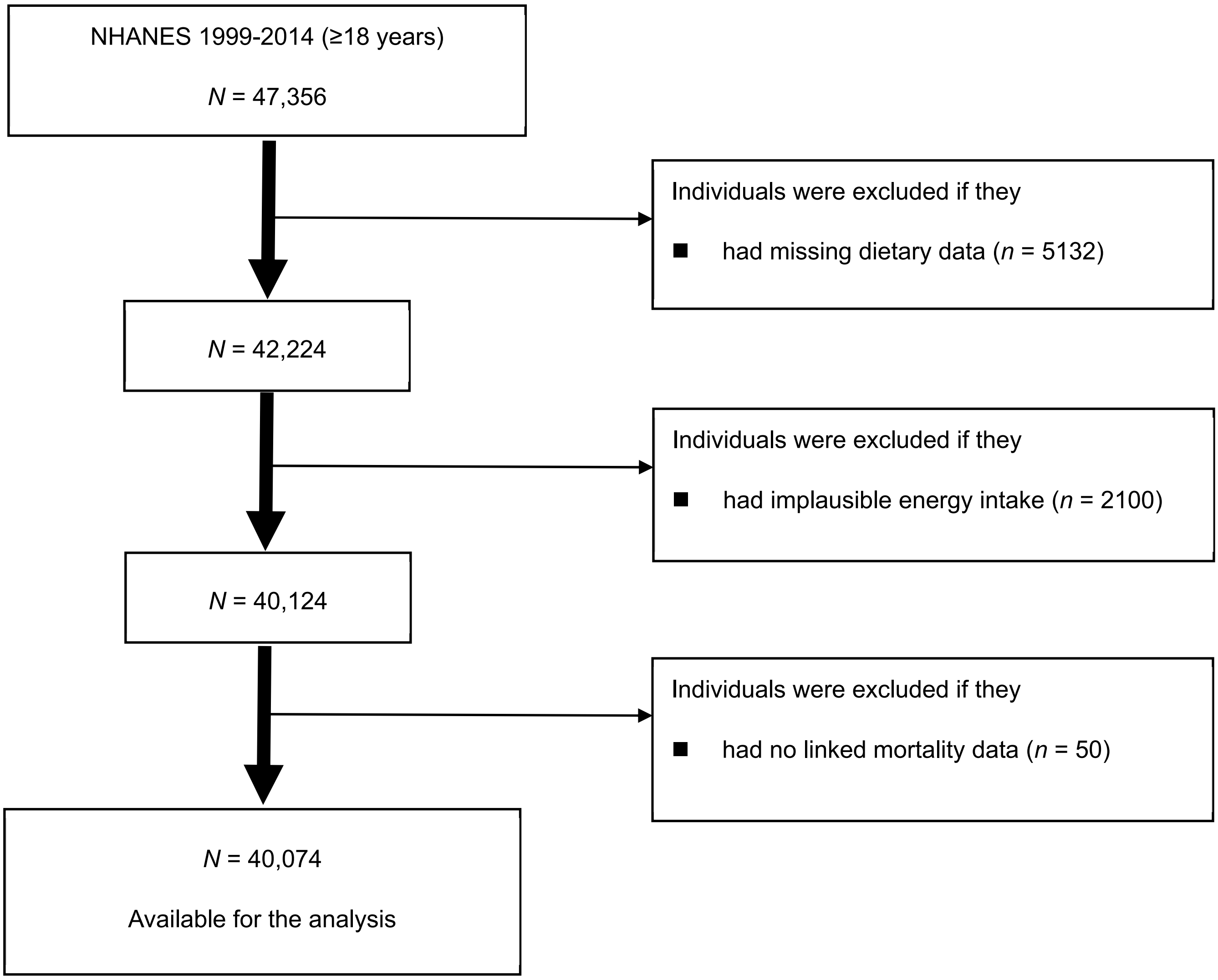

Figure S1. Flow chart of selection of participants in this analysis. NHANES, National Health and Nutrition Examination Survey.

Table S1. Food codes of 100% fruit juice in NHANES (1999-2014)

100% Fruit Juice	Food Code	Description
Grapefruit juice, 100%	61201010	Freshly squeezed
	61201020	NS as to form
	61201220	Canned, bottled or in a carton
	61201225	With calcium added
	61201620	Frozen, reconstituted
Lemon juice, 100%	61204000	NS as to form
	61204010	Freshly squeezed
	61204200	Canned or bottled
Lime juice, 100%	61207000	NS as to form
	61207010	Freshly squeezed
	61207200	Canned or bottled
Orange juice, 100%	61210000	NFS
	61210010	Freshly squeezed
	61210220	Canned bottled or in a carton
	61210250	With calcium added, canned, bottled or in a carton
	61210620	Frozen reconstituted
	61210720	Frozen, not reconstituted
	61210820	With calcium added, frozen reconstituted
Tangerine juice, 100%	61213220	-
Fruit juice blend, 100% juice	61213800	Citrus
	61213900	Citrus, with calcium added
	64100110	-
Cranberry juice blend, 100%	64100200	-
	64100220	With calcium added
Apple juice, 100%	64104010	-

	64104030	With calcium added	
Blackberry juice, 100%	64104600		-
Cranberry juice, 100%	64105400	Not a blend	
Grape juice, 100%	64116020		-
	64116060	With calcium added	
Papaya juice, 100%	64120010		-
Passion fruit juice, 100%	64121000		-
Pineapple juice, 100%	64124020		-
Pomegranate juice, 100%	64126000		-
Prune juice, 100%	64132010		-
Strawberry juice, 100%	64132500		-
Watermelon juice, 100%	64133100		-

NHANES, National Health and Nutrition Examination Survey; NS, Not specified; NFS, Not further specified.

Table S2. Food codes of total fruit juice in NHANES (1999-2014)

Total Fruit Juice	Food Code	Description
Citrus fruit juice	61200500	Acerola juice
	61201000	Grapefruit juice, NFS
	61201010	Grapefruit juice, 100%, freshly squeezed
	61201020	Grapefruit juice, 100%, NS as to form
	61201220	Grapefruit juice, 100%, canned, bottled or in a carton
	61201225	Grapefruit juice, 100%, with calcium added
	61201230	Grapefruit juice, canned, bottled, carton, with sugar
	61201620	Grapefruit juice,100%, frozen, reconstituted
	61201630	Grapefruit juice, frozen, with sugar (reconstituted)
	61204000	Lemon juice, 100%, NS as to form
	61204010	Lemon juice, 100%, freshly squeezed
	61204200	Lemon juice, 100%, canned or bottled
	61204600	Lemon juice, frozen
	61207000	Lime juice, 100%, NS as to form
	61207010	Lime juice, 100%, freshly squeezed
	61207200	Lime juice, 100%, canned or bottled
	61207600	Lime juice, frozen
	61210000	Orange juice, 100%, NFS
	61210010	Orange juice, 100%, freshly squeezed
	61210220	Orange juice, 100%, canned, bottled or in a carton
	61210230	Orange juice, canned/bottled/carton, with sugar
	61210250	Orange juice, 100%, with calcium added, canned, bottled or in a carton
	61210620	Orange juice, 100%, frozen, reconstituted
	61210630	Orange juice, frozen, with sugar, reconstituted with water

61210720	Orange juice, 100%, frozen, not reconstituted
61210730	Orange juice, frozen, with sugar, not reconstituted
61210820	Orange juice, 100%, with calcium added, frozen, reconstituted
61213000	Tangerine juice, NFS
61213220	Tangerine juice, 100%
61213230	Tangerine juice, canned, with sugar
61213620	Tangerine juice, frozen (reconstituted with water)
61213800	Fruit juice blend, citrus, 100% juice
61213900	Fruit juice blend, citrus, 100% juice, with calcium added
61214000	Grape-tangerine-lemon juice
61216000	Grapefruit and orange juice, NFS
61216010	Grapefruit and orange juice, fresh
61216220	Grapefruit and orange juice, canned
61216230	Grapefruit and orange juice, canned, with sugar
61216620	Grapefruit and orange juice, frozen (reconstituted with water)
61219000	Orange and banana juice
61219100	Pineapple-orange-banana juice
61219150	Orange-white grape-peach juice
61219650	Apricot-orange juice
61222000	Pineapple-grapefruit juice, NFS
61222200	Pineapple-grapefruit juice, canned, bottled or in a carton, NS as to sweetened or unsweetened
61222220	Pineapple-grapefruit juice, canned, bottled or in a carton, unsweetened
61222230	Pineapple-grapefruit juice, canned, bottled or in a carton, with sugar
61222600	Pineapple-grapefruit juice, frozen (reconstituted with water)

	61225000	Pineapple-orange juice, NFS
	61225200	Pineapple-orange juice, canned, NS as to sweetened or unsweetened; sweetened, NS as to type of sweetener
	61225220	Pineapple-orange juice, canned, bottled or in a carton
	61225230	Pineapple-orange juice, canned, with sugar
	61225600	Pineapple-orange juice, frozen (reconstituted with water)
	61226000	Strawberry-banana-orange juice
Fruit juice, excluding citrus	64100100	Fruit juice, NFS
	64100110	Fruit juice blend, 100% juice
	64100120	Ambrosia juice
	64100200	Cranberry juice blend, 100% juice
	64100220	Cranberry juice blend, 100% juice, with calcium added
	64101010	Apple cider
	64104010	Apple juice, 100%
	64104030	Apple juice, 100%, with calcium added
	64104050	Apple juice, with added vitamin c
	64104090	Apple juice with added vitamin c and calcium
	64104150	Apple-cherry juice
	64104200	Apple-pear juice
	64104450	Apple-raspberry juice
	64104500	Apple-grape juice
	64104550	Apple-grape-raspberry juice
	64104600	Blackberry juice, 100%
	64104610	Blueberry juice
	64105400	Cranberry juice, 100%, not a blend
	64105500	Cranberry-white grape juice mixture, unsweetened
	64116010	Grape juice, NFS

64116020	Grape juice, 100%
64116030	Grape juice, with sugar
64116040	Grape juice, low calorie sweetener
64116050	Grape juice, NS as to sweetened, with added vitamin c
64116060	Grape juice, 100%, with calcium added
64116100	Grape juice, unsweetened, with added vitamin c
64116150	Grape juice, with sugar, with added vitamin c
64120010	Papaya juice, 100%
64121000	Passion fruit juice, 100%
64122030	Peach juice, with sugar
64123000	pear-white-grape-passion fruit juice, with added vitamin c
64124010	Pineapple juice, NS as to sweetened
64124020	Pineapple juice, 100%
64124030	Pineapple juice, with sugar
64124060	Pineapple juice, unsweetened, with vitamin c
64124200	Pineapple-apple-guava juice, with added vitamin c
64125000	Pineapple juice-non-citrus juice blend, unsweetened, with added vitamin c
64126000	Pomegranate juice, 100%
64132010	Prune juice, 100%
64132020	Prune juice, unsweetened
64132030	Prune juice, with sugar
64132500	Strawberry juice, 100%
64133100	Watermelon juice, 100%
64134000	Fruit smoothie drink, made with fruit or fruit juice only (no dairy products)
64134015	Fruit smoothie, with whole fruit, no dairy
64134020	Fruit smoothie, with whole fruit, no dairy, added protein

	64134025	Fruit smoothie, with whole fruit, non-dairy
	64134030	Fruit smoothie juice drink, no dairy
	64134100	Fruit smoothie, light
	64134200	Fruit smoothie, bottled
Fruit juice and fruit juice mixtures baby food	67202000	Apple juice, baby food
	67202010	Apple juice, with added calcium, baby food
	67203000	Apple-fruit juice blend, baby food
	67203200	Apple-banana juice, baby food
	67203400	Apple-cherry juice, baby food
	67203450	Apple-cranberry juice, baby food
	67203500	Apple-grape juice, baby food
	67203510	Apple-grape juice, baby, with calcium
	67203600	Apple-peach juice, baby food
	67203700	Apple-prune juice, baby food
	67203800	Grape juice, baby food
	67203900	Mango-grape-pear juice, baby
	67204000	Mixed fruit juice, not citrus, baby food
	67204100	Mixed fruit juice, not citrus, with added calcium, baby food
	67205000	Orange juice, baby food
	67211000	Orange-apple-banana juice, baby food
	67212000	Pear juice, baby food
	67230000	Apple-sweet potato juice, baby food
	67230500	Orange-carrot juice, baby food
	67250100	Banana juice with low-fat yogurt, baby food
	67250150	Mixed fruit juice with low-fat yogurt, baby food
	67260000	Fruit juice and water drink, with high vitamin c and added calcium, baby food

NHANES, National Health and Nutrition Examination Survey; NS, Not specified; NFS, Not further specified.

Table S3. HRs (95% CIs) for mortality risk according to each 100 g/day increase in intake of 100% orange juice and 100% citrus juice in NHANES (1999-2014)

Cause of death	HR (95% CI)	
	100% orange juice	100% citrus juice
**All-cause mortality**		
Model 1[a]	1.04 (0.97-1.10)	1.03 (0.97-1.10)
Model 2[b]	1.07 (1.02-1.11)	1.07 (1.02-1.11)
**CVD mortality**		
Model 1[a]	1.00 (0.91-1.10)	0.99 (0.90-1.08)
Model 2[b]	1.04 (0.96-1.12)	1.03 (0.95-1.10)
**Heart disease mortality**		
Model 1[a]	0.97 (0.88-1.08)	0.96 (0.87-1.07)
Model 2[b]	1.00 (0.92-1.09)	0.99 (0.91-1.08)
**Cerebrovascular disease mortality**		
Model 1[a]	1.13 (1.00-1.26)	1.11 (0.99-1.24)
Model 2[b]	1.18 (1.05-1.32)	1.16 (1.04-1.30)

CIs, Confidence intervals; CVD, Cardiovascular diseases; HRs, Hazard ratios; NHANES, National Health and Nutrition Examination Survey.

[a]Covariates adjusted in the model 1 were the same as those in model 1 in **Table 2** (see **Table 2** footnote);

[b]Covariates adjusted in the model 2 were the same as those in model 2 in **Table 2** (see **Table 2** footnote).

Table S4. Sensitivity analyses on association between consumption of fruit juice with the risk of all-cause mortality in NHANES (1999-2014) [a]

	Fruit juice (g/day)					P_{trend}[e]
	0	1 to 124	125 to 249	≥250	Per 100 g/day increase	
**100% fruit juice (g/day)**						
Sensitivity analysis I[b]						
No. of participants	17,655	4,105	3,194	2,626		
No. of deaths/person-years	1,503/115,115	449/26,796	372/21,868	212/18,620		
HR (95% CI)	1 (Reference)	1.18 (1.01-1.37)	1.13 (0.93-1.37)	1.27 (1.04-1.56)	1.07 (1.03-1.11)	0.002
Sensitivity analysis III[c]						
No. of participants	22,662	3,859	3,277	3,560		
No. of deaths/person-years	1,770/192,704	315/28,678	294/26,598	250/32,278		
Model	1 (Reference)	1.22 (1.02-1.47)	1.13 (0.93-1.38)	1.28 (1.01-1.61)	1.07 (1.03-1.11)	0.001
Sensitivity analysis IV[d]						
No. of participants	26,212	4,602	3,984	3,968		
No. of deaths/person-years	2,360/221,503	450/34,355	477/32,111	309/35,819		
Model	1 (Reference)	1.09 (0.93-1.28)	1.17 (0.98-1.38)	1.19 (0.98-1.45)	1.05 (1.01-1.09)	0.010
**Total fruit juice (g/day)**						
Sensitivity analysis I[b]						
No. of participants	17,271	4,130	3,338	2,841		
No. of deaths/person-years	1,479/112,382	452/26,984	379/22, 800	226/20,234		
HR (95% CI)	1 (Reference)	1.17 (1.00-1.35)	1.12 (0.93-1.35)	1.25 (1.02-1.52)	1.06 (1.02-1.11)	0.003
Sensitivity analysis III[c]						
No. of participants	22,117	3,881	3,444	3,916		
No. of deaths/person-years	1,737/187,480	316/28,885	305/27,992	271/35,900		
Model	1 (Reference)	1.21 (1.00-1.46)	1.13 (0.93-1.36)	1.24 (0.99-1.57)	1.06 (1.02-1.10)	0.002

Sensitivity analysis IV[d]						
No. of participants	25,605	4,626	4,179	4,356		
No. of deaths/person-years	2,312/215,722	453/34,533	492/33,768	339/39,765		
Model	1 (Reference)	1.09 (0.93-1.27)	1.13 (0.96-1.38)	1.16 (0.97-1.41)	1.04 (1.01-1.08)	0.026

CIs, Confidence intervals; HRs, Hazard ratios; METS, Metabolic equivalent tasks; NHANES, National Health and Nutrition Examination Survey.

[a]Covariates adjusted in the models were the same as those in model 2 in **Table 2** (see **Table 2** footnote);

[b]Sensitivity analysis upon the exclusion of individuals who had a single 24-hour dietary recall;

[c]Sensitivity analysis within individuals who were free of any history of congestive heart failure, angina pectoris, coronary heart disease, heart attack, stroke, cancer, or diabetes at baseline;

[d]Sensitivity analysis upon the exclusion of individuals who died within 3 years after dietary assessment;

[e]Linear trend test was conducted by treating 100% or total fruit juice as continuous variable in the model.
